# Unsung Heroes: Gay Physicians’ Lived Journeys During the HIV/AIDS Pandemic

**DOI:** 10.1177/2158244019827717

**Published:** 2019-02-04

**Authors:** Carl GA Jacob, Daniel Lagacé-Roy

**Affiliations:** 1 University of Ottawa, Ottawa, Ontario, Canada; 2 Royal Military College of Canada, Kingston, Ontario, Canada

**Keywords:** physicians, patients, gay/homosexual, HIV/AIDS, trajectories/experiences/journeys, epidemic/pandemic

## Abstract

The HIV/AIDS pandemic was a major crisis at the end of the 20th and beginning of the 21st century. Such a defining moment in the history of health-related infections led to transformations in its proponents, as well as their medical practice. This research article, using a study consisting of semi-structured interviews with six Canadian gay physicians from different Canadian HIV/AIDS treatment centers, aims to offer insights into their lived journeys, from 1981 to 2009, while they attempted to treat, care for, and cure/heal their gay HIV/AIDS patients. The results of the study, deduced from a qualitative and interpretative data analysis, suggest that through reflection on their experiences during the HIV/AIDS pandemic, they transformed their personal and professional identities, and rethought their relationship with their patients, as well as their professional, pharmaceutical, and community networks. These results are testimonies from Canadian gay physicians who fought against the HIV/AIDS pandemic and who advocated for their gay HIV/AIDS patients. In fact, these results are evidence of an untold and valuable period in medical history. For some, it will serve as a reminder. For others, it will be foreign. It was a time marked by a major crisis that mobilized gay militant physicians who were personally and professionally affected, and who were forever transformed by their response to the HIV/AIDS pandemic. This is their hereto untold lived journeys.

## Introduction

The human immunodeficiency virus/acquired immune deficiency syndrome (HIV/AIDS) pandemic (pandemic) was a major crisis at the end of the 20th and beginning of the 21st century. Such a defining moment in the history of health-related infections led to transformations in its proponents, as well as their medical practice. This research article aims to offer insights into the lived trajectories/experiences (journeys) of six Canadian gay physicians while they attempted to treat, care for, and cure/heal (treat) gay HIV/AIDS patients from 1981 to 2009.

According to Valdiserri (2011), in June 1981, a major event, referred to as a tempest, crisis, lethal threat, or new outbreak (among other terms), shocked the world and baffled the medical establishment (Bayer & Oppenheimer, 2000; Finkelstein, 2018; Joint United Nations Programme on HIV/AIDS, 2002; Lévy & Quévillon, 2014-2015; Piot & Quinn, 2013; U.S. Department of Health and Human Services, 2018). An unknown virus, disease, or infection was reported for the first time by the Centers for Disease Control and Prevention (CDC) in the *Morbidity and Mortality Weekly Report* (U.S Department of Health and Human Services, 2018). It was first called gay cancer or gay plague by the media, and gay-related immune deficiency (GRID) by the CDC (Altman, 1982; Chibbaro, 1982; Cichocki, 2009; Kher, 1982). In 1982, the CDC renamed the infection AIDS.^1^ It was not until 1986 that HIV was termed (Aldrich & Wotherspoon, 2001). The infection soon grew into an epidemic, as it was prevalent within a larger community, mostly gay (Bayer & Oppenheimer, 2000; Ungvarski, 2001). As cases appeared in other parts of the world, besides North America, such as Europe and Africa, it was eventually recognized as a pandemic (Altman, 2011; Smith, 2001).

Throughout the more than four decades since the appearance of the HIV infection, many narratives have been published (e.g., Alderman & Frey, 1997; Boulanger & Thomas, 2008; McCoy, 2005; Scannell, 1999; Smith, 2001). They speak of “courage, cowardice, hope, despair, compassion, and bigotry” (Ungvarski, 2001, p. 26). They describe the fear of the unknown, the spread of the disease, death, sexuality, and alternative lifestyles, such as bisexuality, homosexuality, transgender, and drug use (Witt Sherman, 2000; Witt Sherman & Ouellette, 2000). Because of the attitudes, beliefs, interpretations, opinions, perceptions, and values that individuals held about HIV/AIDS, many social problems arose, such as stigmatization, discrimination, and prejudice (De Oliveira Almeida, Vandenplas-Holper, & De Ketele, 1992; Witt Sherman, 2000; Witt Sherman & Ouellette, 2000). Moreover, because of the convergence of the myriad of uncertainties regarding HIV prevention, HIV/AIDS treatment, and opportunistic infections, many HIV-infected patients developed comorbidities with the above-mentioned issues or fears. Furthermore, some health professionals “succumb[ed] to hysteria and abandon[ed] their objectivity and compassion in the care of patients with HIV/AIDS” (Dunkel & Harfield, 1986, p. 114; Witt Sherman, 2000; Witt Sherman & Ouellette, 2000).

In response to the shock provoked by the sudden and still unknown infection, as well as the hysteria, abandonment, and victimization of some HIV-infected gay members by their community, “the gay, lesbian, bisexual, and transgender (LGBT) communities quickly came to realize that if they didn’t take action, nobody would” (U.S. Department of Health and Human Services, 2018, p. 3). Many gay health professionals, and gay physicians in particular, embraced the same view. They responded to the call to action because they had friends or family members who were HIV-infected, or as a personal response to the abandonment and rejection of their patients who were either gay, or HIV-infected, or both simultaneously (Pascreta & Jacobsen, 1989). By heeding the call to action, these physicians modified their public image and, at the same time, transformed their personal identity. They came out of their professional sexual neutrality and became “openly” gay physicians, profoundly implicated in the political, social, and medical causes of their patients, friends, and community (Jacob, 2012).

Unfortunately, over time, memories of what it was like to meet head-on a grim, contagious, disfiguring, lethal, and sexually transmitted threat like HIV/AIDS have begun to fade. It was a “time when medicine was all but powerless” (Bayer & Oppenheimer, 2000, p. 3) and when “people with HIV [/AIDS] were fired from their jobs, kicked out of their apartments, denied health care and abandoned by their families” (AIDS Legal Council of Chicago, 2013, p. 4). However, those who survived this period appear to be ready to speak about it again. There is a new generation of activists, archivists, artists, historians, and researchers, among others, who were born during the HIV/AIDS pandemic, who now need to make sense of this grim period (Finkelstein, 2018). Moreover, there is a growing number of archival projects, books, documentaries, and gallery exhibitions that are now devoted to the topic, with more underway.

One such undertaking is this scientific research article which presents an unpublished study conducted between 2008 and 2009 with six Canadian gay HIV/AIDS physicians (study). Their stories are compelling journeys that transformed their personal and professional identities, and their medical practice. While it aims to offer insights into the lived journeys of unsung heroes (physicians), it also serves as a reminder of their courage as lobbyists and advocates (militants) for their HIV/AIDS patients (patients) during the pandemic.

### Literature Review

At the time of the study, only two types of literature pertaining to the study’s topic, that is, The Use of Experiential Learning in the Transformation of the Medical Practice of Six Professionals in the Context of the HIV/AIDS Pandemic,^2^ were available. They included books written by several nonmedical authors setting the study’s context, as well as autobiographies written by medical and nonmedical authors providing a firsthand account of physicians’ lived journeys. These two types of literature served to establish a preliminary understanding of the patients’ trajectories from the onset of the infection to the patients’ death. They also provided an account of physicians’ journeys from the onset of the infection to their patients’ death and beyond, including attending their funerals, for example, at different key periods of the pandemic’s trajectory. These key periods were identified as follows: *Period 1*, HIV/AIDS Emergence; *Period 2*, Recognized Pandemic and Militancy Rise; and *Period 3*, HIV Chronicity and Militancy Decrease (see appendix—HIV-AIDS Pandemic Trajectory: Three Periods). A few examples of the books and autobiographies from nonmedical and medical authors are highlighted here for their significant input into the study.

In their book titled *Good Doctor Good Patients: Partners in the HIV Treatment*, Rabkin, Remien, and Wilson (1994) present patients’ and their physicians’ beliefs about the characteristics of and expectations from the physician–patient therapeutic relationship. They discuss medical and psychological challenges faced by both physicians and patients from the initial HIV/AIDS diagnosis up to and including the end-stage of the disease, and the dying process. They also describe specific skills and strategies that physicians can use to meet these challenges and discuss the role that friends, family, and professional counselors play in addressing the ongoing challenges of living with HIV/AIDS. In *Reports From the Holocaust: The Story of an AIDS Activist*, Kramer (1994) shares his views as an AIDS activist and advocate, as well as on AIDS and LGBT civil rights from the beginning of the pandemic. In addition, he documents his time spent working in community organizations against HIV/AIDS (e.g., Gay Men’s Health Crisis and ACT UP) and beyond. As for Shilts (1988), in his book titled *And the Band Played On: Politics, People and the AIDS Epidemic*, he discusses the discovery and spread of HIV/AIDS and comments on the government’s indifference and political infighting during the early 1980s.

In his autobiography titled *To the Friend Who Did Not Save My Life*, Guibert (1993) shares his own journey on his AIDS-related physical and psychological suffering, and reveals his physician–patient therapeutic relationship. Hodge-Wright (2004), in her autobiography titled *Life Lessons: Stories of Hope, Love and Laughter in the Face of AIDS*, discloses stories of love, betrayal, forgiveness, and joy from the counseling experiences which impacted her life. She also reveals her efforts to find meaning in every positive and negative life experiences, including when individuals are confronted with AIDS. As for Whitmore (1988), in *Someone Was Here: Profiles in the AIDS Epidemic*, he tries to humanize the notions of illness, epidemic, and courage by reaching out to men and women whose lives were changed by the HIV/AIDS epidemic. In fact, these autobiographies, and many more, present the human toll of the HIV/AIDS pandemic (see also Bluestein, 1997; Callwood, 1988; Cox, 1990; Gélinas-O’Meara, 1994; Kavanagh, 2007; Monette, 1988; Oyler, 1988).

In his autobiography titled *The Least of These My Brethren: A Doctor’s Story of Hope and Miracles on an Inner-City AIDS Ward*, Baxter (1997) shares the life stories of suffering and redemption of HIV/AIDS patients. These stories capture the hope and miracles he encountered while attempting to treat HIV/AIDS patients during his three and a half years in a New York AIDS center. In *My Own Country: A Doctor’s Story of a Town and Its People in the Age of AIDS*, Verghese (1985) tells a story of the AIDS epidemic inside a hospital in rural America while confronting his own prejudices and fears. In *Strong Shadows: Scenes From an Inner City AIDS Clinic*, Zuger (1995) presents stories of HIV/AIDS patients she attempted to treat by illustrating their struggles with infections and medications, as well as the effects on them and their families. As for the book titled *AIDS Doctors: Voices From the Epidemic. An Oral History*, authors Bayer and Oppenheimer (2000) recount the stories of 73 physicians whose lives were centered on the HIV/AIDS pandemic (see also Boulanger & Thomas, 2008; Cyr, 2001; McCoy, 2005; Olivier, 1996; Scannell, 1999; Selwyn, 1998).

### Themes

The books and autobiographies mentioned in the literature review helped to identify the themes needed for the preparation of the semi-structured interview grid and the creation of the initial conceptual taxonomy that was used to organize and analyze data. The following themes emerged initially, as they were frequently mentioned and emotionally charged. Physicians highlighted the impact of the emergence of HIV/AIDS, their recognition of the epidemic and the pandemic on them, their patients, and their profession. They mentioned, among other themes, the uncertainties generated by a multiplication of scientific and medical discoveries/findings, as well as the developments in the treatment of opportunistic infections related to the HIV infection. Physicians also talked about the perceptions they and other physicians held about sexuality, drug use, and HIV/AIDS. Finally, they disclosed how they modified the way they practiced medicine at the outset of their careers, moving from a physician-centered practice toward a more patient-centered practice.

## Study Methodology

The data presented in this research article were gathered through the descriptive, interpretive, and exploratory qualitative case study conducted by Jacob (2012). The case study methodology approach was chosen by Jacob because it helped researchers learn about the global meaning of the physicians’ experiences in the context of the pandemic through detailed descriptions pertaining to their lived journeys. It was also preferred because the study was situated at the heart of physicians’ everyday life while interacting with and attempting to treat their patients. Finally, it was privileged because the HIV/AIDS pandemic is a contemporary event with facts and meaning related to the physicians’ journeys inserted into their professional–patient relationships.

The study conducted by Jacob (2012) identified six Canadian gay HIV/AIDS dedicated physicians’ experiential learning and captured their transformations during the moments they recalled while attempting to treat their patients. It also explained the processes they used and the motivations that compelled them to learn from their experiences and transform their personal and professional identities, as well as their practice. Jacob outlines how they rethought their relationship with their patients, other professionals, and community networks during those same gripping moments.

The approach adopted when recruiting physicians for that study was purposive because it selected as participants Canadian gay dedicated HIV/AIDS physicians who met specific criteria (Bernard, 2002; Lewis & Sheppard, 2006). These criteria were inspired by the criteria used by patients when choosing to consult a physician during the pandemic (Jacob, 2012). They mainly sought out physicians who could and were willing to provide information by virtue of their expertise and competence in sexually and blood-transmitted infections and in the HIV/AIDS prevention and treatment field. They looked for those who were involved and had experience, since the beginning of the pandemic, in the treatment of patients, and who had the reputation of having been confronted by the devastating effects of the pandemic throughout its trajectory by the HIV/AIDS research community. Moreover, they sought out physicians with the same sexual preference as theirs and identified as being gay, as well as individuals who possessed a militancy background, that is to say, mobilized and advocated for their patients and their community. Finally, patients migrated toward locations where these specific physicians had established their practice in large Canadian urban centers, mainly in private and public health clinics specialized in the treatment of patients with sexually and blood-transmitted infections, and in the HIV/AIDS prevention and treatment field.

An invitation to participate in semi-structured interviews was sent via email to a pool of physicians who met the above selection criteria. Their credentials and reputation were well-known by the HIV/AIDS research community through prior research studies conducted with them and/or through the physicians’ presentations at national and international conferences (e.g., Canadian Conference on HIV/AIDS Research, and the International AIDS Conference). Six physicians responded to the invitation, thus forming the six cases. Their median age was 48 years old and their experience in the treatment of HIV/AIDS patients spanned an average of 19 years, with their medical practice consisting of 200 HIV/AIDS patients, in mean terms. Finally, the six physicians practiced medicine in large Canadian urban centers: four in private health clinics and two in public health clinics specialized in the prevention, treatment, and care of patients with sexually and blood-transmitted infections (e.g., HIV).

The following sections explain the interview process, as well as the data collection, production, and analysis as it is suggested by many specialists in the case study methodology (Hlady, 2002a, 2002b; Karsenti & Savoie-Zajc, 2000; Miles & Huberman, 2005). After the standard procedures for qualitative studies, that is, explanation of the study, interview procedure, and consent form, the second phase of study began, that is, data collection through interviews. Interviews were conducted either at the physician’s workplace (e.g., office) or in a room on the university campus. They were to last roughly 1 hr. However, the physicians’ generosity would not be dampened by a time constraint! The interviews were conducted using an interview grid, that is, closed-ended questions for quantitative information gathering (e.g., age, number of years of experience, number of HIV/AIDS patients, and work environment) and open-ended questions for qualitative information gathering about the physicians’ lived journeys. Besides being audio-taped, a researcher journal was maintained to capture the researcher’s impressions and perceptions following each interview. The study used the period that included the start of the epidemic (around 1981) through to the said infection chronicity (around 1998), as well as up to the interview period, 2008-2009.

Data were produced using the complete audio-taped transcription of each interview assisted by “word processing software.” To allow for a preliminary analysis, the complete transcription of the audio tape for each interview was performed within 24 hr following the interview.

Based on Merriam (1988), as well as her peers (Hlady, 2002a, 2002b; Karsenti & Savoie-Zajc, 2000; Miles & Huberman, 2005), the data analysis steps included, in a successive and cumulative manner, reading over the interview transcript (verbatim) and researcher journal after each interview session, to develop a certain familiarity with the data; creating headings manually and using a data processing software (e.g., Atlas-ti); and indicating recurrent ideas, that is, notions, concepts, themes, and so on. This process was used after each interview to readjust the interview grid so that new information could be obtained where gaps were identified. This back-and-forth movement between data collection and analysis was maintained throughout the data collection phases.

During the data analysis for each transcript, an iterative process was used to separate the data into the following categories: units of meaning, coding, categorization, and established relationships between them (e.g., commonalities, differences, and patterns) (Jacob, 2012). Following the last interview, an in-depth data analysis was conducted for each transcript and between transcripts.

The study’s scientific rigor and trustworthiness were established (Merriam, 1988) through a process which included peer reviews (the research team) of every interview transcript, discussions of the research process, and presentations of the data’s interpretation. If any modifications to the established research protocol were needed, they were suggested and agreed upon by the research team before implementation. Finally, physicians who were interviewed were asked to review the draft data interpretation of their interview for their comments and suggestions. Two physicians presented their comments and suggestions.

## Results

The following two broad-meaning categories emerged from the data analysis of the semi-structured interviews conducted during Jacob’s (2012) study: namely, *Category 1*: the transformation of the physicians’ identities, both personal and professional; and *Category 2*: the transformation of the relationships physicians nurtured with their patients (e.g., physician–patient therapeutic relationship) and with various networks (e.g., professional, pharmaceutical, and community networks). These categories are explained and illustrated in the following sections.

### Category 1: Transformation of the Physicians’ Identities: Personal and Professional

Following the growth of the pandemic, physicians reported noticing the transformation of the perspective they held of their personal and professional identities. At the personal level, physicians were led to reconsider and alter their medical practice. For some physicians, this meant, first, that they would devote more time to the treatment and care of patients from the gay community to which they belonged and, second, that they would demonstrate their commitment to and solidarity with their community by advocating and lobbying personally on their behalf. In other words, at the personal level, these physicians were involved not only as physicians but also as openly gay physicians.

. . ., we got involved . . ., that was our involvement (Doctor Antoine). (p. 150)

. . ., at the start of my career, I was always open about my sexuality, but then, I was openly gay. I was no longer scared of being an openly gay physician . . . There were many gay physicians who decided to get involved in the treatment of HIV-infected gay patients. We took control of our patients. We became their spokesperson (Doctor Denis).^3^ (p. 150)

At the professional level, a paradigm shift took place concerning their role. First, they became committed to the development of professional knowledge related to medications (drugs) and treatments. In Doctor Claude’s words, “We participated in the development of almost all new drugs” (p. 151). They started conducting research protocols with the patients’ voluntary participation. Therefore, they went from being medical practitioners to being medical researchers practitioners.

Second, physicians were called upon to play other roles besides the traditional doctor role, such as social worker, psychologist, guide, counselor, and trainer, as well as educator.

At that time, we were not only physicians . . .; we were also social workers (Doctor Antoine). (p. 156)

. . . even if you are a doctor, all you can be, is a guide. . . . In many cases, we are also the psychological support, the moral support (Doctor Denis). (p. 156)

. . . our role is to offer advice (Doctor Edgar). (p. 156)

. . . I am a guide. I am a trainer for my patients (Doctor François). (p. 156)

I take care of . . . their medical education . . . I tell them to protect themselves and others with whom they are having unprotected sex. I talk to them about the potential risks to themselves, such as being contaminated by another HIV strain during unprotected sex. I then talk to them about the possible impacts that they were not aware of, one day, that they may have transmitted the HIV infection to someone else. My reaction was to do some sex education (Doctor Claude). (p. 167)

Because of the unusual high occurrence of death among their patients, physicians faced disorienting dilemmas that led them to modify some of the perceptions that they held about their medical practice. The following are examples of various dilemmas they encountered.

#### A dilemma about their perception of death and time

This dilemma takes into account their felt inability to do anything within their medical practice to reduce the infection’s occurrence or mitigate its psychological and physical effects on their patients.

We saw what it was like to lose patients, as we lost many. My worst episode was when I lost fourteen patients in ten days. It was awful. It meant that when a patient who entered your office was sick, you would send him to the hospital. The next patient was the friend of the previous patient. He would tell you that the previous patient died. And him as well, you would send him to the hospital and he would die. . . . It was like that non-stop. Afterward, it was the hospital staff who would call you to tell you that one of your patients died. . . . I learned to live one day at a time (Doctor Antoine). (pp. 152-153)

There was this kind of fatalism in the air non-stop (Doctor Bernard). (p. 152)

What we told patients was that they were going to die in a few months, or in a few years, if all went well. Therefore, it was a very dark era (Doctor Claude). (p. 152)

Like everyone’s life, it is the length that is the difference (Doctor Denis). (p. 153)

#### A dilemma about their perception of euthanasia

This dilemma led them to consider, at the risk of their own career, the demands made by their patients, their patients’ friends, or their relatives, to assist them in dying so they may die in dignity.

What are they [the patient’s friends] going to do with what I gave them? Are they going to cause the patient’s death? At the same time, I was thinking, if the patient is not well, maybe it is better that he dies. But at the same time, I was aware that euthanasia was illegal (Doctor Claude). (p. 153)

#### A dilemma about their perception of risk

Physicians realized that they would incur risk in exercising their profession as long as lasted the scientific and medical uncertainties, at the time of the pandemic, concerning the way that the infection was transmitted, often placing themselves in situations that could possibly endanger their own health and life.

I was scared for my own health every time I was in contact with a patient because at that time, I did not know the modes of transmission (Doctor Claude). (p. 154)

There was no reason why I could not fall sick to this disease. At the beginning, I didn’t know if I could catch it while touching a patient. I never stopped being close to them. But, I didn’t know if, by accident, I would get infected and what impact it would have on me (Doctor Denis). (p. 154)

#### A dilemma about their perception of aggressive and futile therapies

Physicians realized that they should cease to impose their will on their patients and begin to determine when they should stop therapies, treatments, and medications as they did not improve the patient’s health.

At a certain moment, I realized that I had to stop wanting to cure a patient at all cost. As long as I thought that there was a chance that I could cure the patient, I would try other medications. I would behave this way because when I would succeed in healing a patient, it was well perceived by other doctors (Doctor Denis). (p. 154)

I thought that it was important to realize that there was a limit to how much medication I could administer to a patient when the medication was no longer efficient to cure the disease (Doctor Edgar). (p. 154)

#### A dilemma about the traditional images that physicians held about places to practice medicine

Physicians became aware that they had to go back and forth between private clinics, local community health centers and hospitals, and less conventional places to treat their patients, such as shelters, homes and hospices, private residences, and prisons.

We monitored patients at home. . . . We went to . . . shelters for AIDS patients (Doctor Denis). (p. 156)

I also worked in a prison where some of my patients were (Doctor Claude). (p. 156)

#### A dilemma about their physical and professional limitations

In a context where the number of HIV/AIDS cases was constantly increasing, physicians became acutely aware of their limitations as their patients were dying, and they neither knew the virus that infected them nor the many opportunistic diseases that were affecting them.

I am not all mighty . . . (Doctor Claude). (p. 157)

. . . a doctor can do a lot, but there are limits to what we can do. We cannot stop everything, death or the infection. It is sometimes difficult to change the course of the infection (Doctor Edgar). (p. 157)

#### A dilemma about scientific and medical certainties

Physicians were practicing in an environment where medication was more and more numerous and constantly changing (Garfield, 1993), where data (evidence-based) to support the decisions they were making (e.g., on the start and stop of treatment) were missing, and where the knowledge about the medication they were prescribing and its food interactions was not available.

Often, I would tell my patients that the drug they are taking now has a specific value in time. Possibly one day, this drug will be completely obsolete (Doctor Antoine). (p. 159)

When I did not have information on the interaction between a drug I was prescribing and certain foods, such as garlic, I would tell my patients that it was better to be careful and not to eat it every day (Doctor Antoine). (p. 159)

#### A dilemma about their perception of the Faculty of Medicine and the College of Physicians

Physicians recognized the limits of the training they received from the Faculty of Medicine, as well as the College of Physicians’ poor understanding of the physicians’ daily realities when facing this new medical situation that required, among other, alternative practices.

At the Faculty of Medicine, we did not learn to manage a large number of deaths and moreover a large number who were young (Doctor Antoine). (p. 159)

In the field of complementary and alternative medical therapies, doctors do not have much training (Doctor Edgar). (p. 160)

#### A dilemma about medicine in general

According to these physicians, the medical profession was still paternalistic (Emanuel & Emanuel, 1992), often blaming patients for their mishaps and sustaining prejudice against the gay community.

Medicine is quite paternalistic. The doctor in his medical practice is quite paternalistic. . . . in the medical domain, patients are often blamed for what happens to them (Doctor Edgar). (p. 160)

I was in a system that was old school. You could be gay, but you could not show it. You did not invite your lover to suppers at the Department of Medicine. You went with a girl, you went alone, or you didn’t go at all (Doctor Denis). (p. 160)

### Category 2: Physician–Patient, Professional, Pharmaceutical, and Community Network Relationships

Following the growth of the pandemic, physicians mentioned perspective transformations in the physician–patient therapeutic relationship, patient empowerment, and medical therapeutic conversations, as well as in their professional, pharmaceutical, and community network relationships.

In their medical practice, physicians were compelled to transform the physician–patient therapeutic relationship because of the significant role social movements against HIV/AIDS played in HIV/AIDS information dissemination to its membership, and the growing number of informed and empowered patients they treated. This situation compelled physicians to adopt a more egalitarian therapeutic relationship. In fact, they started engaging in sustained and challenging therapeutic conversations with their patients. Physicians realized that they could not actively continue to confront, judge, or even ignore them. Therefore, they had to explain the decisions they made regarding their patients’ health and pay more attention to their choice of words when having to provide advice, discuss options, and negotiate treatments and medications, as well as the start or cessation of a treatment and/or medication.

When you treat those patients, it is more stimulating. When you tell those patients to take such and such a drug, they ask you why they must take that one and not another one. You must explain your decision (Doctor Antoine). (p. 207)

I explain more my train of thought and the drug that I am going to prescribe when I treat those patients. I take more time to explain how I arrived at that decision (Doctor Bernard). (p. 207)

What I learned is that it is important to be careful not to be confrontational with those patients. . . . I am sometimes in a situation where I must learn to negotiate with those patients, for example, when to start or to end a treatment (Doctor Claude). (pp. 168, 207)

We do not judge patients (Doctor Antoine). (p. 168)

What I learned is that I must . . . respect the patient who got infected and who lives with the HIV infection (Doctor Denis). (p. 168)

Physicians also modified their medical practice to strengthen the criteria surrounding privacy, while continuing to comply with the requirements prescribed by law (mandatory reporting), to address the new patients’ demand (non-disclosure).

The doctor-patient relationship is one that stays between four walls. Very often, we are the only ones who know that someone is HIV-positive. Their workplace doesn’t know it. Their friends don’t know it (Doctor Antoine). (p. 165)

The patient did not want the reason for his death mentioned on his death certificate (Doctor Denis). (p. 165)

Physicians realized that the border (therapeutic distance) between themselves and their patients was gradually blurring as more and more of the patients who sought their HIV/AIDS expertise had often been their friends, lovers, colleagues, or co-gay community members.

It was a clientele to which I identified a lot with because these men were like me, at that time, young gay men who were discovering themselves a little, who were starting to live their gay life (Doctor Claude). (p. 165)

The doctor-patient border was not very strong. Often there was none. I began to see friends at the clinic . . . And I remember when a great friend of mine, a work colleague, came to see me at the clinic (Doctor Denis). (p. 166)

Moreover, physicians often had a “history” with these individuals. They were their patients before they were diagnosed, up to their death and beyond, as many physicians started attending their patients’ funerals, a rare occurrence before the pandemic.

I am close enough to my patients. . . . I developed a stronger bond with some of them. When you have been treating them for over twenty years, you develop a stronger bond with them (Doctor Claude). (p. 166)

It was important that I be there, when HIV was discovered, during the period of opportunistic infections, and at the end of the HIV trajectory, to be able to say goodbye. I attended my patients’ funerals (Doctor Denis). (p. 166)

Through this renewed physician–patients therapeutic relationship, physicians developed a greater understanding of their patients’ distress/helplessness. Moreover, they became cognizant of their non-inhibited sexual behaviors.

My patients tell me everything, their intimacy, the emotions related to their sex life, their preferred drugs, and their cruising habits. . . . A patient started telling me that he went to the sauna . . ., where everyone, it seems, goes to have sex. It is not well looked upon for someone to put on a condom in that sauna (Doctor Claude). (pp. 170-171)

A patient told me that he had had many partners who did not protect themselves . . . (Doctor François). (p. 170)

Patients would tell me about the rave parties they attended . . . (Doctor Denis). (p. 170)

They also learned about their patients’ rejection, on a regular basis. In Doctor Edgar’s words, “Patients would tell me about the rejection they face from society in general and from the gay community in particular . . .” (p. 171).

At some point during the pandemic, some physicians understood that they had to start rebuilding the boarder (therapeutic distance) which had once existed between themselves and their patients to be more effective and efficient. In fact, this therapeutic distance was essential in repositioning the physicians’ role, in changing certain habits, or to educate their patients on health, sexuality, and blood-transmitted infections, among other topics.

. . . physicians had to start rebuilding the therapeutic distance, a distance between a doctor and the infection of his patients, the distance between a doctor and his patients . . . (Doctor Bernard). (p. 167)

Physicians also noted and understood the potential role reversal that could take place. At times, the traditional physician–patient role was reversed when patients became aware that their physician was possibly more ill (e.g., workload or HIV-infected) than they were, or when they were simply concerned about their physician’s physical and mental well-being.

. . . it felt like the patient was almost trying to comfort me . . . (Doctor Claude). (p. 168)

. . . it was the patient who was taking care of the doctor. As if he thought that the situation was too intense for the doctor (Doctor Denis). (p. 169)

#### Professional, pharmaceutical, and community network relationships

The data showed that physicians were also obligated to transform the way that they viewed and dealt with other key networks. They had to develop new contacts with professionals who were sympathetic to their cause, fighting HIV/AIDS (e.g., pharmacists and medical specialists), not only to find technically relevant information but also to obtain reliable and supportive collaborations.

. . it was really through those years that we learned which doctors were sympathetic to our cause. They were the doctors who were not prejudicial to our patients. We developed a network, people we knew, teams or specialists to whom we had easier access. . . . I had to let go of control to enable the specialists to do what needed to be done (Doctor Denis). (pp. 173-174)

The neurologist who evaluated the patient was very good with patients who were HIV-infected (Doctor Antoine). (p. 173)

Their professional networks were also extended to pharmaceutical companies. However, they needed to modify the perception they held about them, from an industry whose primary focus was profit maximization to one whose focus was more humanitarian, focused on patients. They also needed to realize that the pharmaceutical industry was moving from an industry reliant on universities and private sector medical research and development to one more reliant on physicians for the same purpose.

If you have a new patient who does not yet have access to the drugs he needs, the pharmaceutical companies are there to help. I once phoned all the pharmaceutical companies to get access to a drug before it was available because I had a patient who was really sick. . . . It was possible (Doctor Edgar). (p. 174)

. . . if we hadn’t participated in that research protocol, if we didn’t have a structure that allowed us to do research, which is not always possible in a private clinic, our patients would have had to wait three years. Some would have died because of the lack of access to medication. Therefore, it was fantastic to save all those patients . . . (Doctor Claude). (p. 175)

Finally, physicians also had to add to their networks gay community organizations, as they were also involved in the prevention and fight against HIV/AIDS. In fact, these organizations became partners and information disseminators, which often came directly from the physicians themselves.

. . . activists, we knew them well. We kept in contact, with the gay community organizations against HIV and we worked with them (Doctor Denis). (p. 177)

We knew the information that they had in that organization, because it was us, the group of doctors, who wrote the articles circulating there. We work in close collaboration with them. They are very useful (Doctor Antoine). (p. 177)

I used, for a long time, the services like those offered by that organization or those support groups. . . . I did many information sessions directly to patients, thanks to that organization . . . (Doctor Claude). (p. 177)

To sum up, the data showed that the physicians transformed their personal and professional identities, as well as their practice to take into account the growing number of HIV-infected patients they treated who were empowered and informed, because of the social movements against HIV/AIDS that supported them.

They also transformed the relationships they nurtured with their patients (e.g., physician–patient therapeutic relationship) and with various networks (e.g., professional, pharmaceutical, and community networks).

## Discussion

The following section brings together three types of findings for discussion. The first section compares differences between the themes that emerged from the study and those that resulted from the analysis of the previously mentioned autobiographies. The second one presents new findings obtained through a recent literature review. Finally, the third section shows that some themes raised by this research article still persist today (recurring themes).

### Differences

The themes (identified in the autobiographies that were used to prepare the semi-structured interview grid and to create the initial conceptual taxonomy that was used to organize and analyze data) are almost a mirror image of the themes that emerged from the data analysis of the interviews. These themes highlighted the impact of the emergence of HIV/AIDS; emphasized the uncertainties generated by a multiplication of scientific and medical discoveries/findings; talked about the perceptions physicians held about sexuality, drug use, and HIV/AIDS; and have drawn attention to the way they practiced medicine, going from a physician-centered practice toward a patient-centered practice.

However, the following themes emerged from the data of the study of some Canadian gay HIV/AIDS dedicated physicians’ journeys but did not appear in the physicians’ autobiographies. Under the first category mentioned in the study, the transformation of the physician’s personal and professional identities, the study brought to light the militant implication of these gay physicians as they advocated and lobbied for their HIV/AIDS gay patients. As they recognized themselves as being gay physicians, they reconsidered and altered their involvement in their patients’ medical, political, and social causes/battles (e.g., treatment, medication, and rights). In doing so, they demonstrated their solidarity to the gay community. To strengthen their ties with their community, they also modified their medical practice by treating their patients in conventional (e.g., private clinics, community health centers, and hospitals) and unconventional work environments, namely, in their patients’ residences/homes.

The study also underlines that during the pandemic, these gay physicians recognized that pharmaceutical companies had acquired a more open attitude regarding their participation in research protocols. Thereafter, a paradigm shift took place: from medical professionals they became professional-practitioner researchers. They became committed to the development of professional knowledge related to medications (drugs) and treatments.

Finally, as they were constantly confronted with a fatal disease, they recognized that there might be a place and a time where euthanasia would be appropriate, even though it was still illegal at the time.

Under the second category mentioned in the study, the transformation of the physician–patient relationship, as well as the professional, pharmaceutical and community networks, the study brought to light how gay HIV/AIDS dedicated physicians made more place for discussion about the HIV/AIDS patients’ lifestyle (e.g., sexuality, homosexuality, cruising, raves, drugs, and saunas). They also modified their therapeutic conversations by being less paternalistic, refraining from blaming patients for what happened to them, and by not discriminating and stigmatizing their patients. While doing so, they also blurred the traditional border that existed between themselves and their patients, such as by going to their patients’ funerals. They built networks of professionals who were sympathetic to their causes/battles and developed links with social movements to increase the support networks for their patients. They became directly involved in gay community organizations against HIV/AIDS and learned to play an active role as openly gay informants and activists to disseminate information about HIV/AIDS.

The results from the study, unlike the physician’s autobiographies, make no mention of the role spirituality or religion played while patients were facing a life-threatening and limiting infection such as HIV/AIDS, neither did it mention the reflections patients and physicians provided in relation to it. Yet, the place of spirituality and religion in the lives of patients facing a life-threatening and limiting infection such as HIV/AIDS and their physicians is often mentioned in the autobiographies examined for the study (e.g., Baxter, 1997; Boulanger & Thomas, 2008; Scannell, 1999; Verghese, 1985).

Then, one day, tired of seeing my friends and patients who were only 25, 30, 35 years old die one after the other from AIDS, I questioned my faith and felt anger towards God. So, I looked at the sky and, whether God exists or not, I spoke to him that day. I asked him, “Why is this happening? Why is everyone I love dying? If you existed, you would not be doing this. It’s too unfair.” (Boulanger & Thomas, 2008, pp. 59-60)

### New Findings

Through a recent literature review pertaining to gay nurses caring for gay HIV-infected patients, three research papers surfaced about physicians who attempted to treat patients in the context of the pandemic. The first one, by Witt Sherman and Ouellette (2000), is titled *Physicians Reflect on Their Lived Experiences in Long-Term Care*. The second one, by Murbach (1991), speaks about *Médecins et patients au temps du sida: le cas de Montréal*; and the third one, by Lévy and Quévillon (2014-2015), addresses the *Pratiques professionnelles médicales et VIH/sida: des témoignages à la fiction romanesque*. However, they never openly breach the topic from the same specific angle as Jacob’s study, namely, the lived experiences of gay HIV/AIDS dedicated physicians attempting to treat gay HIV/AIDS patients.

Witt Sherman and Ouellette (2000) present the physicians’ reflection on “their professional histories in caring for patients with AIDS” (p. 278). Even though the physicians they interviewed included one gay physician, the data were not segregated using that variable. This research article’s interest resides in the additional information pertaining to the following categories that are described below.

#### Reasons for entering the AIDS care frontline

These physicians adopted the AIDS care domain as a natural progression of knowledge and skills as they had “prior experience and knowledge in virology, epidemiology, or infectious disease” (p. 278), and because it was at the “cutting edge of medicine” (p. 279). They also mentioned that they chose that domain of work because they felt that “there was a heightened sense of connection with themselves, their patients, and the personal and professional values and beliefs that guided their lives and medical practices” (p. 279).

#### Rewarding aspects of AIDS care

Once physicians had learned to deal with the numerous stressors and emotional consequences of AIDS care, they felt that “the rewards in caring for patients with AIDS outweighed the stressors” (p. 281). The rewards that they mentioned included “developing long-term, reciprocal relationships with patients, developing partnerships in care, experiencing personal and professional growth, and a sense of gratification” (pp. 281-282).

#### Aspects of the physician–patient therapeutic relationship and patient characteristics that promote a willingness and commitment to care

Physicians mentioned that many aspects of the physician–patient therapeutic relationship and patients’ characteristics may influence a physician’s willingness, desire, and commitment to attempting to treat patients, such as “‘patients who demonstrate self-care,’ ‘patients who trust my [their] care,’ ‘patients who value our [their] relationship,’ and ‘patients who teach us [them] how to die with grace and dignity’” (p. 283).

#### Recommendations offered by physicians specializing in the AIDS care domain

Physicians offered many recommendations to their colleagues considering the AIDS care domain as a future profession. Such examples include working on caring (e.g., empowering patients, listening to them, and talking to them in a language they can understand), learning to turn mistakes into lessons (e.g., using reflection on action, and realizing that one is human), being realistic yet optimistic (e.g., admitting that one doesn’t have all the answers, and dealing with uncertainty about the patient’s condition with honesty), and learning to say no when you need to protect oneself (e.g., valuing and augmenting one’s personal relationships) (Witt Sherman & Ouellette, 2000).

#### Lessons learned

Sometimes, physicians learned more from what was not said than from what was said. In fact, they realized that there was no “justice in this epidemic” (p. 284), and as such, the virus did not discriminate. Such realization helped them to accept patients the way they were and, more importantly, that all patients deserved to be treated and cared for, and to receive quality care. As there was no mention of God or spirituality—like in Jacob’s study—in either “causing the epidemic, or alleviating the suffering” (p. 284), physicians seem to have chosen this path of life based on “conscious choices,” and to be dedicated to their field for “humanistic values” (p. 284). However, such a life choice and dedication did not prevent them from burning out, experiencing depression, or marital conflicts.

Murbach (1991) presents a study conducted in Montreal, in 1989-1990, where the data were not segregated using the variable of homosexuality. It attempted to comprehend how physicians and patients perceive their relation, the disease and death, and care and medical research. The interest of this research resides in the fact that it mentions experiences not previously accounted for in other studies.

#### Medical personnel obligations

During the pandemic, a large societal debate was underway to determine the medical personnel’s obligations, and, in particular, the physician’s obligation to attempt to treat patients, as well as their freedom to choose their patients.

#### Reasons physicians entered the AIDS care domain

Like Witt Sherman and Ouellette (2000), Murbach (1991) provides reasons why physicians would decide to specialize in the AIDS-care domain. They perceived that they would have access to interesting medical cases, as AIDS was a major scientific event that could bring them peer recognition. Finally, some new specialists considered the AIDS domain as a means to access research funds and consolidate their research notoriety.

#### Cultural differences and alternative medicine

It was noted that most physicians adopted a degree of openness to diverse alternative medicines, and even supposedly “miracle medications” (p. 77), but without severing bridges with traditional medicine. However, there might have been a cultural bias in looking for such alternatives, as Francophone physicians were more prone to have confidence in “natural” treatments then their Anglophone counterparts.

#### Honesty in the context of research trials

The need to be “very” honest (p. 80) with HIV patients was more than necessary, as new research trials had a “50/50 chance” of success (p. 80). In this sphere of uncertainty, physicians, as well as patients, were desperate to try something—anything new. They had to be honest to themselves, as physicians, and their patients needed to be at the forefront of these research trials because they had never been done before.

Lévy and Quévillon (2014-2015) present a paper that triangulates sociological, autobiographies, and novel narratives to extract the transformations of the medical practice, the place of the affect related to the illness and death, the physician–patient therapeutic relationship, and its representation during the pandemic’s evolution.

The data extracted from these narratives were not segregated using the variable of homosexuality. This research is interesting because it speaks about the emotional sphere in terms of its connection to the disease, death, and the relationship with their patients.

#### Affective challenges

The emotional sphere mentioned above speaks directly to the ambivalent feelings physicians experienced while attempting to treat their patients. While these feelings are comparable to those mentioned by patients, such as rage, agony, dissension, sadness, acceptance, revolt, and hope, they also include compassion, fear, and culpability.

Through the same literature review, two books also surfaced: one by Dr. Johnson, titled *Working on a Miracle*, explaining his combat against the HIV infection and his search for a treatment, and the other by Dr. Waddell, called *Gay Olympian: The Life and Death of Dr. Tom Waddell*, describing his life quests (e.g., “coming out,” a career, a family), as well as his battle against the infection.

In his book, Johnson (1997) describes his odyssey and “apparently successful fight against HIV” (Campbell, 1997, p. 1). Four years earlier, “an autopsy on an HIV-infected corpse” (p. 1) went wrong. When his scalpel slipped, he went from being a doctor to being a patient (Johnson, 1997).

The interest of this book resides in the fact that Johnson formulated his “battle plan to fight the disease” (Campbell, 1997, p. 1), which “ran contrary to conventional treatments” (p. 1). It consisted in immediately starting an aggressive treatment, at a time when the tendency was not to start treatment immediately, of a “veritable alphabet soup of drug therapies” (p. 1) which included 28 pills per day.^4^

Johnson did not allege having found a cure for AIDS. In fact, at the time of Johnson’s discovery, other AIDS experts,

Warn[ed] against unrealistic optimism, fearing the disease may be dormant or hiding out in another part of his body, such as the lymph nodes. (Campbell, 1997, p. 2)

Twenty-one years later, Dr. Johnson is still working as a pathologist and a Professor at the University of Rochester School of Medicine & Dentistry in Rochester, New York.

Finally, Waddell and Schaap’s (1996) book presents, in the first person, the trials and tribulations of a teenager realizing that he might be gay, and his subsequent coming out, as a gay physician, once he had full-blown AIDS. This book is interesting because it is written by a gay physician who passed away from an AIDS-related infection. Waddell used his book to transmit to his daughter, and to the world, his vast knowledge, using his extraordinary ability to teach and translate knowledge. Waddell explains the full chronology of the progression of the infection and his attempts to cure/heal it. He also gives explicit examples of his lifestyle before and during the epidemic, up until his death. The book also confirms that Waddell, like some gay physicians, wrote “articles on the subject for the California *Voice*, and I am trying [tried] to bring some common sense to the crisis and offer [offered] people ways to begin to alter their behaviour in the least traumatic way” (p. 33). There was discrimination, stigmatization, and prejudice toward AIDS victims but, as Waddell points out, there were also poignant reunions with past lovers and friends because of the disease. His book also eloquently presents the state of panic that was present, at that time, in Castro’s gay community, in San Francisco, California.

Waddell also talked about how the gay community had to evolve to stay alive. “Those who do not change their sex practices will die” (p. 169). “In this era of AIDS, it’s sensible to be monogamous or even celibate” (p. 180). He also mentioned how he was “inundated with ‘cures’ from friends and strangers—some bizarre, some logical, all ineffective” (pp. 204-205, 220). Spirituality, or the lack thereof, was also commented on by the author in the following manner, before closing his eyes for the last time, “Well this should be interesting” (p. 223).

Without a doubt, these narratives, and the information they contain, would have positively impacted Jacob’s (2012) study, by providing more information in the preparation of the interview grid, the categorization for data analysis, and more opportunity for disclosure.

### Recurring Themes

Some themes raised by this research article still persist today (recurring themes). Among them are the absence of services to key populations, discrimination and stigmatization, the lack of regulatory framework, and the importance of communication in the physician–patient therapeutic relationship. In 2011, the World Health Organization (WHO) acknowledged that health services to key populations are often not tailored to or integrated into other health and social services, nor do they meet the needs of key populations in terms of their values, desires, or preferences. As well, they do not address their practical constraints.

Further in the report, WHO recognized that structural barriers to health services, such as stigmatization and discrimination, continue to exist and they must be removed to improve the knowledge of “serostatus” in sex workers, men who have sex with men, transgender people, and drug injection users (key populations), while promoting and protecting human rights and promoting access to health services.

In the same report, WHO highlighted that many countries lack a regulatory framework, such as laws, policies, regulations, or interventions to protect human rights, to enable the rapid regulatory approval of new and generic medicines and diagnostics, and to expedite their marketing approval.

Finally, in 2014, a study by Young reminded us of the importance of a physician–patient therapeutic relationship based on communication.

. . . optimal health requires attention to provider-patient communication across the entire HIV care cascade. While care cascade metrics begin with testing and end with viral suppression, healthy aging with HIV requires attention to aspects of health and healthcare communication. (p. 1)

These recurrent themes converge toward one central theme that is the patient’s needs. It is through the patient’s needs that health and social services are recognized and should be improved; that discrimination and stigmatization happen and should be addressed; that human rights are challenged and should be fought; and that communication with physicians occurs and should be evaluated.

## Research Implications

It is important to emphasize that the study conducted by Jacob in 2012, and on which this research article is based, revealed research implications that are equivalently associated with the patients’ needs, the medical practice, and various stakeholders. A closer look at these research implications shows that the treatment of patients, especially in time of crisis, challenges the traditional evidence-based medicine and provokes new practices. As seen in this research article, these practices, based on lived journeys, have produced new scientific knowledge that increases the professional–patient relationship and the value of the patient who becomes more involved in the process of healing (patient informant and patient empowerment).

The lived journeys of these six Canadian gay physicians will serve the medical community and the public health domain when they attempt to treat, care for, and cure/heal patients who are infected with future diseases. This is especially true where at first, little institutionalized, evidence-based, knowledge is available and patient stigmatization and discrimination is prevalent, for instance, the severe acute respiratory syndrome (SARS), the human infected H1N1 influenza, and the mosquito-borne ZIKA. This reference to past outbreaks will help today’s physicians approach these new diseases with a more human touch and a view to the possibility of being transformed personally and professionally (e.g., militancy and researcher practitioner). It will also encourage them to remain open to modified relationships (e.g., a new approach to their patients as informants and trainers, their colleagues, other professionals, and community organizations) that could help them in their battle against the infection.

## Other Research Interests

Because of the interest created by Jacob’s (2012) study, as well as the research papers and books that surfaced since, we are now moving our research interest from gay physicians to gay nurses’ lived journeys while caring for gay HIV-infected patients during the HIV/AIDS pandemic. This new research article’s interest lies in the fact that nurses have an increased proximity with patients; therefore, they may have had similar or different lived experiences with gay HIV-infected patients than their gay physician counterparts.

Our literature review has already uncovered one book written by a gay nurse and one written by a lesbian nurse (Czerwiec, 2017; Varsalone & Deering, 2016). No scientific papers using gay nurses as subjects have surfaced yet. However, one book and a few articles were found with nurses caring for HIV/AIDS patients as subjects (Bennett, 1992; McGarrahan, 1994; Witt Sherman, 2000).

## Conclusion

The objective of this research article was to provide the lived journeys of Canadian gay HIV/AIDS dedicated physicians in the context of the HIV/AIDS pandemic in an effort to draw a better picture of what it was like to be a gay physician during that particular period. The research background was based on books and autobiographies that set the stage for the HIV/AIDS context. In addition, they provided an account of the physicians’ trajectories/journeys/experiences as they to treat, care for, and cure/heal HIV-infected patients throughout the pandemic.

In the context of the HIV/AIDS pandemic, the Canadian gay physicians’ personal and professional identities and the way they viewed their practice were transformed by their lived journeys. They became openly gay HIV/AIDS dedicated and militant physicians, advocating and lobbying for their patients. They altered their physician–patient therapeutic relationship, making it more open and egalitarian. They modified their relationship to their networks, namely, community organizations, other colleagues, pharmacists, and the pharmaceutical industry, by increasing their personal and professional involvement in gay community organizations against HIV/AIDS, building a network of professionals able and willing to care for their patients, and becoming physician researchers practitioners.

The data presented and the results interpreted offered a candid insight into the lives of Canadian gay HIV/AIDS physicians, at a time when the medical world was not prepared for such a devastating disease. Moreover, this research has highlighted issues that were critical to bringing changes in the way physicians saw their medical practice. The therapeutic relationship between physicians and patients is a good example of this type of change, as both, physicians and patients, had to re-define what to expect from each other.

As people read these physicians’ lived journeys, they are reminded of a time when gay physicians who practiced during the HIV/AIDS pandemic were militants, advocated for their gay HIV/AIDS patients, and lobbied for a cure. This article lifted the veil of darkness that shrouded the contribution made by these unsung heroes, shining of spotlight on their selfless dedication to their patients in the hope that readers will recognize their sacrifices and come to realize that they were personally and professionally affected and forever transformed because of their involvement in their attempts to treat, care of, and cure/heal gay HIV-infected patients during the HIV/AIDS pandemic.

Finally, according to Finkelstein (2018), these narratives are a reminder that the full “story of [HIV/] AIDS cannot be written yet [until a] cure, curative, functional cure, or vaccine” (pp. 219-220) against HIV/AIDS is found, there is a need to re-count the way it was and how it is now translated into our present.

## Appendix

**Appendix:** HIV-AIDS Pandemic Trajectory: Three Periods.

**Period 1—HIV/AIDS Emergence** Patients: Gay individuals who contracted the HIV infection – Isolated and destitute – Little or badly informed Medical Practitioners: Generalists – Little solidarity with patients – Generally little militancy activities – Interested by knowledge, but little engagement in new required knowledge production Gay Social Movements: Against HIV – Non-existent or non-visible
**Period 2—Recognized Pandemic and Militancy Rise** Patients: Gay individuals who contracted the HIV infection – Militancy rise – Active search for information – Participation in conferences, seminars, and workshops Medical Practitioners: Gay generalists – New medical practice mode (experimental medicine, experiential learning used) – Committed to knowledge production, not only its use – Institutionalized knowledge and traditional continuing medical education modes challenged – Patient solidarity, militancy Gay Social Movements: Against HIV – Visions and alternative medical knowledge production – Provides members with a range of services and training
**Period 3—HIV Chronicity and Militancy Decrease** Patients: Gay individuals who contracted the HIV infection – Militancy and empowerment decline – Conferences, seminars, and workshop participation decline Medical Practitioners: Gay generalists – Initial and continuing medical education integrate new knowledge (knowledge institutionalization) – Medical practice centered mainly on the preservation of health and the cure of diseases – Experimental medicine continues, but less engaged in knowledge production – HIV infection increasingly considered chronic (an “implicit expectation” that infected individuals will survive the HIV infection) Gay Social Movements: Against HIV – Fewer militancy organizations – Have difficulty recruiting volunteers – Focused on specialized service provision

## Notes

1. According to the University of California in San Francisco, a diagnosis of AIDS is made whenever a person is HIV-positive, the CD4+ cell count is below 200 cells per microliter, their CD4+ cells account for fewer that 14% of all lymphocytes, or they have been diagnosed with one or more of the AIDS-defining illnesses (infections, cancers, or neurological disorders).

2. The exact title is as follows: Recours à l’apprentissage expérientiel dans la transformation de la pratique médicale de six professionnels dans le contexte de la pandémie du VIH-Sida (see Jacob, 2012).

3. All citations presented in the results section of this article have been loosely translated from French to English.

4. 6 Ritonavir, 3 Saquinavir, 2 gel caps of AZT and 1 gel cap of 3TC at breakfast and at supper; plus 1 gel cap of AZT, 1 gram of vitamin C, a cocktail of vitamin B and one gel cap of beta-carotene at bedtime.
